# Formation Mechanism of Characteristic Flavor Substances in 3-Year-Old Diannan Small-Ear Pig Ham: Lipidomics and Flavoromics Study

**DOI:** 10.3390/foods14173098

**Published:** 2025-09-04

**Authors:** Wenli Tao, Zhenzhu Li, Guangqiang Wei, Yue Wang, Yuzhu Wang, Wenbin Zhang, Chenghao Zhang, Yunmei Chai, Huaming Mao, Yufang Li, Aixiang Huang

**Affiliations:** 1 College of Food Science & Technology, Yunnan Agricultural University, Kunming 650201, China; 2 College of Animal Science & Technology, Yunnan Agricultural University, Kunming 650201, China

**Keywords:** ham, lipids, volatile flavor compounds, key metabolites

## Abstract

Diannan small-ear pig (DSP) ham is known for exceptional flavor. However, the composition of flavor components and the mechanisms underlying flavor development remain unclear. In this study, we employed lipidomics, flavoromics, and ultra-high-performance liquid chromatography–tandem mass spectrometry technologies to investigate the composition and formation mechanism of DSP ham flavor compounds. On a 10-point scale, the results demonstrated that DSP ham had good flavor qualities with sensory scores of 8.6 ± 0.52 for flavor, 7.9 ± 0.57 for taste, 8.2 ± 0.79 for texture, 8.8 ± 0.42 for color, and 8.3 ± 0.48 for acceptability. A total of 1534 lipids, 80 volatile flavor compounds, and 25 free amino acids were identified in the ham, including 14 characteristic lipids and 28 characteristic flavor compounds. Triglycerides (TG) and diacylglycerol (DG), two important lipids, are broken down into free fatty acids, which are essential building blocks for flavor formation. Non-volatile sweet amino acid L-alanine and bitter amino acid L-lysine are combined with volatile components, including 1-octene-3-ol, hexanal, benzaldehyde, and octanal, to enhance the development of DSP ham flavor. Correlation analysis indicated that key lipids, including TG, DG, and phosphatidylcholines (PC), facilitate the formation of volatile compounds in DSP ham via the glycerophospholipid metabolic pathway. This study provides a theoretical reference for further research and product development of high-quality DSP ham.

## 1. Introduction

Ham, a widely consumed meat product, is generally prepared using pickling, fermentation, and air-drying methods. The distinct flavor significantly impacts the market value and consumer approval of ham, comprising both volatile and non-volatile flavor substances [1]. The production of volatile flavor compounds is mainly due to lipolysis, lipid oxidation, proteolysis, Maillard reaction, Strecker degradation, and other biochemical reactions during the curing process of ham. The length of the maturation period significantly influences the concentration of volatile compounds in the final product, resulting in a more pronounced flavor and aroma [2]. Traditional hams have been shown to contain about 200 important volatile chemicals, such as alcohols, ketones, carboxylic acids, esters, hydrocarbons, and aldehydes [3]. In addition, non-volatile flavor compounds such as acids and lipids are crucial for the final taste of ham as lipids act as the main precursors for the synthesis of meat aroma. Most volatile compounds formed in cooked ham arise from the oxidative breakdown of oils or their interaction with Maillard reaction products. The initial phase in transforming lipids into taste compounds is hydrolysis, which significantly elevates the concentration of free fatty acids that are subsequently oxidized to generate several volatile chemicals. Free fatty acids mainly arise from phospholipids, which are consequently seen as essential elements in the production of volatile flavor compounds in dry-cured ham [4]. The taste of ham is closely associated with its fat composition. Consequently, analyzing the lipid makeup of ham can provide a comprehensive understanding of the mechanisms underlying ham flavor development.

Lipidomics can identify lipid markers with biological effects to reveal the relationship between ham flavor precursors and flavor. Zhang et al. [5] employed headspace–gas chromatography (GC)–ion mobility spectrometry techniques to analyze the volatile flavor compounds in Jinhua fatty ham (FH) and lean ham (LH). A total of thirty-three volatile flavor compounds were discovered in both FH and LH. The LH exhibited a greater abundance of total alcohols and acids, whereas the FH presented a wider variety of aldehydes, ketones, esters, heterocycles, and sulfur-containing compounds. Li et al. [6] investigated the presence of free amino acids and volatile organic compounds (VOCs) in Xuanwei ham throughout various maturation stages, utilizing a combination of high-throughput sequencing, along with liquid chromatography and gas chromatography–mass spectrometry (GC-MS). A total of 25 distinct amino acids were identified, with the lowest level observed in ham from 3-year-old animals. In addition, 59 types of VOCs were detected, including 17 types of esters; the 4-year-old ham had the highest ester content. Li et al. [7] conducted analyses of lipidomics and volatileomics on lamb meat characterized by varying levels of intramuscular fat content. In positive- and negative-ion modes, 79 of the 842 lipids identified were significantly different between the two groups (variable importance of projection > 1, *p* < 0.05). Yang et al. [8] investigated the relationship between lipid changes and zinc protoporphyrin formation during the processing of Norden ham using lipidomics. Ultra-high-performance liquid chromatography combined with tandem mass spectrometry (UHPLC-MS/MS) was utilized to detect 1002 lipids in positive-ion mode and 470 lipids in negative-ion mode from samples of Norden ham. Chen et al. [9] evaluated the effects of different types of salt substitutions on the degradation of lipids and volatile flavor substances in reconstituted duck ham. A total of 55 volatile flavor substances were identified, comprising 13 aldehydes, 12 alcohols, 3 acids, 1 ketone, 11 esters, 12 alkanes, and 3 other compounds. Throughout the process, there was a consistent rise in the overall quantity of free fatty acids, with the highest levels found in saturated fatty acids, followed by monounsaturated and polyunsaturated fatty acids.

The flavor of ham is influenced by both the breed of pig and the processing methods employed. Traditional pig breeds utilized in ham production include the Wujin, Jinhua, and Dianlu pigs. The Diannan small-ear pig (DSP) is a native Chinese pig breed that is primarily raised in the southern region of Yunnan Province, which typifies the subtropical climate of Yunnan [10]. Moisture, fat, and protein contents of small-ear pork in southern Yunnan were reported to be 69.42–74.65%, 1.28–7.38%, and 20.97–25.04%, respectively, as determined by near-infrared spectroscopy [11]. It is evident that DSP pork is of high quality. The meat of DSP from southern Yunnan is renowned for its exceptional quality, characterized by delicate muscle fibers, uniform fat distribution, and a fresh, tender, waxy texture that retains an authentic pork flavor. This breed, therefore, serves as an ideal material for the production of high-quality ham. In this study, given its outstanding characteristics, DSP has been processed into ham and fermented for 3 years under standardized conditions. The flavor of the ham improved significantly under these conditions, exhibiting tender red and white meat along with a robust salty aroma. However, the characteristic flavor compounds and their formation mechanism of DSP ham remain unclear. To close the research gaps in this area, this study uses sophisticated analytical techniques to further examine the lipids, free amino acids, and volatile taste compounds in 3-year-old DSP hams.

To address this gap, our research utilized untargeted ultra-high-performance liquid chromatography with optical emission mass spectrometry (UHPLC-OE-MS) alongside targeted ultra-performance liquid chromatography multiple reaction monitoring mass spectrometry (UPLC-MRM-MS/MS), combined with gas chromatography–mass spectrometry (GC-MS), to investigate the lipids, free amino acids, and volatile flavor compounds found in three-year-old DSP ham. The overall objectives of this study were to (1) elucidate the lipid mass spectrometry and identify the characteristic lipids, (2) reveal the flavor profile and characteristic flavor substances, and (3) analyze the correlations of free amino acids, lipids, and flavors to determine the flavor formation mechanism of 3-year-old DSP ham. This study closes the research gap on local characteristic pig breeds and offers fresh theoretical support and practical assistance for the quality investigation of DSP ham through multidimensional analysis.

## 2. Materials and Methods

### 2.1. Sample Preparation

The source materials for this study were the hind legs of a 12-month-old DSP. The hind legs weighed 7.7 ± 0.29 kg and measured roughly 50–60 cm in length. The hind legs were carefully cut, followed by the spreading and cooling of the fresh legs. Thereafter, the legs were salted and cured at ambient temperature, sustained between 5 °C and 15 °C, with a humidity range of 70% to 90%. The salt dosage was determined to be 10 kg for each leg and was regulated to roughly 1000 g. The salting procedure was performed six times, following which the salt was permitted to accumulate for a duration of one to four weeks. The salted meat was subsequently cleansed, sun-dried, molded, and suspended in a ventilated space to air-dry and ferment for three years. The result was ham aged three years [12]. Four samples were obtained from four DSP hams that had been cured and fermented for three years, and these samples were then preserved in a refrigerator at −80 °C for subsequent examination.

### 2.2. Main Reagents

Main reagents are 2-Octanol (TCI), alkanes (mixed standard) (ampere spectrum), methanol (Methanol), acetonitrile (Acetonitrile), formic acid (Formic acid), methyl tert-butyl ether (MTBE), ammonium formate (Ammonium formate), dichloromethane (Dichloromethane), and isopropyl alcohol (Isopropanol) (CNW Technologies).

### 2.3. Sensory Analysis

The sensory evaluation was conducted according to the methodology described in Ding et al. [13] with minor modifications. For the sensory evaluation, ten qualified assessors were chosen from Yunnan Agricultural University. (5 girls, 5 males, ages 22–30, with no prior history of olfactory or taste problems). The 3-year DSP hams’ color, flavor, texture, aroma, and general acceptance were assessed. Every participant in the sensory evaluation signed an informed consent form acknowledging their knowledge of the experiment’s potential dangers. Participation in the study was entirely optional, and participants were free to leave at any moment. The biceps femoris of four hams were cut into 2 mm thick slices, and the hams were subjected to sensory evaluation after cooking for 20 min. Sensory assessment: During the assessment of two separate samples, participants had to gargle with 50–60 mL of filtered water at least twice in order to prevent weariness and lingering odor from interfering with sensory rating. The sensory score for each attribute was rated out of 10 as follows: 1–3 points indicate “weak,” 4–6 points indicate “medium,” and 7–10 points indicate “strong.” The evaluation results are presented in the form of a radar chart [14].

### 2.4. Lipidomics Analysis

Lipidomics analysis was performed on a Phenomenex Kinetex C18 column (2.1 mm × 100 mm, 2.6 μm) using a Vanquish (Thermo Fisher Scientific, Waltham, MA, USA) ultra-high-performance liquid chromatograph. Metabolite extraction was performed at low temperature by placing 25 mg DSP ham samples into EP tubes, followed by the addition of homogenized beads and 200 μL water. Subsequently, 480 μL of the extract (methyl tert-butyl ether: MeOH = 5:1 (*v*/*v*); CNW Technologies, Shanghai, China) containing the isotope-labeled internal standard (CNW Technologies) was added. Following vortex mixing for 30 s, the homogenate was placed in a homogenizer (35 Hz, 4 min) and then transferred to an ultrasound ice-water bath for 5 min. This step was repeated three times, and the sample was left to stand at −40 °C for 1 h. The sample was then centrifuged at 4 °C, 3000 rpm (900× *g*, radius 8.6 cm) for 15 min, and 300 μL of the supernatant was taken and dried in an EP tube at low temperature with vacuum. A 200 μL extract (dichloromethane methanol: MeOH = 1:1, *v*/*v*) was added to the dry sample for 30 s, followed by ultrasound in an ice-water bath for 10 min. The sample was centrifuged at 4 °C, 12,000 rpm (13,800× *g*, radius 8.6 cm) for 15 min, and then 175 μL of the supernatant was injected into the vial for analysis.

The lipid analysis was performed using a C18 (Thermo Fisher Scientific, Waltham, MA, USA) liquid chromatography column (2.1 mm × 100 mm, 2.6 μm) with an Orbitrap Exploris 120 mass spectrometer. The mobile phase A comprised a solution of 40% water and 60% acetonitrile, augmented with 10 mmol/L ammonium formate (CNW Technologies). For phase B, a solution comprising 10% acetonitrile and 90% isopropanol was formulated, also incorporating a 10 mmol/L aqueous solution of ammonium formate (CNW Technologies). A typical injection volume of 2 μL was utilized. The MS parameters were as follows: sheath gas flow rate: 30 Arb; aux gas flow rate: 10 Arb; capillary temperature: 320 °C (positive) or 320 °C (negative); full MS resolution: 60,000; MS/MS resolution: 15,000; collision energy: 15/30/45 in NCE mode; spray voltage: 3.8 kV (positive) or −3.4 kV (negative).

ProteoWizard software 3.0 was used to convert the original mass spectrum into mzXML format. XCMS was used for retention time correction, peak identification, peak extraction, peak integration, peak alignment, and other quality control steps; minfrac was set to 0.5, and the cutoff was set to 0.3. Identification of lipids was conducted utilizing our proprietary library, which is built upon XCMS software V1.51.0, a custom R package V3.0, along with the LipidBlast database.

### 2.5. Analysis of Free Amino Acid Content

DSP ham samples were placed in EP tubes, two small steel balls were added, and 1000 μL extract (acetonitrile:methanol: water at a 2:2:1 volume ratio and isotope-labeled internal standard mixture precooled at −40 °C) was added to the sample and vortexed for 30 s. The sample underwent grinding at a frequency of 40 Hz for a duration of 4 min, which was followed by ultrasonic treatment lasting 5 min in a bath of ice water; this procedure was repeated three times. The samples were placed at −40 °C for 1 h and then centrifuged at 4 °C, 12,000 rpm (13,800× *g*, radius 8.6 cm) for 15 min; 100 μL of the supernatant was transferred to the liquid chromatography injection bottle for UPLC-MS/MS analysis.

The corresponding amount of the standard was accurately weighed in a 10 mL volumetric flask, and a standard stock solution of 10 mmol/L was prepared. The corresponding amount of the standard stock solution was then placed in a 10 mL volumetric flask to prepare a mixed standard solution. The standard solution was diluted to obtain a series of calibration solutions (including an isotope-labeled internal standard mixture with a concentration consistent with that in the sample). The free amino acid content of DSP ham was analyzed by a Waters ACQUITY UPLC BEH (Waters Corporation, Shanghai, China) amide liquid chromatography column (100 × 2.1 mm, 1.7 μm, Waters). Phase A was a 1% formic acid aqueous solution, and phase B was 1% formic acid acetonitrile. The column oven temperature was 35 °C, the sample tray was set to 4 °C, and the injection volume was 1 μL. The MS parameters were as follows: capillary voltage of +4000/−3500 V, nozzle voltage of +500/−500 V, gas (N_2_) temperature of 300 °C, gas (N_2_) flow of 5 L/min, sheath gas (N_2_) temperature of 250 °C, sheath gas flow of 11 L/min, and nebulizer at 45 psi.

Before UPLC-MS/MS analysis, the standard solution of the target compound was introduced into the mass spectrometer. For each target compound, several transition ion pairs with the highest signal intensity were selected, and the MRM parameters were optimized. The ion pairs that exhibited the optimal response were chosen for quantitative analysis, while the remaining ion pairs were utilized for the qualitative analysis of the target compound. The Agilent MassHunter Work Station Software (B.08.00, Agilent Technologies, Santa Clara, CA, USA) and DATA DRIVEN FLOW V2.0 were utilized for all steps related to the acquisition of MS data and the quantitative analysis of the target compounds.

### 2.6. Analysis of Volatile Flavor Compounds

GC-MS was used to analyze the volatile flavor compounds of DSP ham. Samples (800 ± 16 mg) were weighed in a 20 mL headspace vial, and 10 μL of the 2-octanol internal standard solution (TCI; 10 mg/mL stock in H_2_O) was added. In a separate headspace injection bottle, 10 μL (50 mg/L) of n-alkanes mixed standard (ampere spectrum; CNW Technologies) was added. The GC-MS parameters were as follows: extraction temperature (incubation temperature) of 60 °C, preheat time of 15 min, extraction time (incubation time) of 30 min, desorption time of 4 min, front inlet mode set to splitless mode, front inlet septum purge flow of 3 mL/min, helium as the carrier gas, DB-Wax column (30 m × 250 μm × 0.25 μm), and a column flow rate of 1 mL/min. The oven temperature was ramped to 40 °C, held for 4 min, raised to 245 °C at a rate of 5 °C/min, and held for 5 min. The front injection temperature was 250 °C, the transfer line temperature was 250 °C, the ion source temperature was 230 °C, and the quad temperature was 150 °C. The ionization voltage (electron energy) was −70 eV, the mass range (*m*/*z*) was 20–400, the system was run in scanning mode, and the solvent delay was 2.37 min.

### 2.7. Calculation of Relative Odor Activity Value (ROAV)

This research clarifies the mechanism of flavor development in 3-year-old DSP ham through an analysis that examines the relationships between lipids, free amino acids, and volatile flavor compounds.

The detection rate of compounds in each sample group was calculated, and the compounds with a detection rate greater than 50% were retained. The coefficient of variation (CV) of the peak area of the compounds in each sample group was calculated, and the compounds with a CV of less than 30% were retained. After the above two screening steps, the relative percentage content (*C*%) of the average peak area of the compounds in each sample group was calculated. Compounds with a flavor perception threshold annotation were retained; the *ROAV* of the compound that had the greatest contribution to the overall flavor of the sample group (named *ROAV_stan_*) was defined as 100; the *ROAVs* of the other compounds (named *ROAV_A_*) were then calculated with the following formula:

$${ROAV}_{A}\approx 100\times \frac{{C\%}_{A}}{{C\%}_{stan}}\times \frac{T_{stan}}{T_{A}}$$

where *C*%*_A_* is the relative percentage content of the average peak area of other compounds, *C*%*_stan_* is the relative percentage of the average peak area of the compounds that had the greatest contribution to the overall flavor of the sample group, *T_stan_* is the sensory threshold of the compound with the greatest contribution to the overall flavor of the sample group, and *T_A_* is the sensory threshold of other compounds.

The *ROAV* of all compounds was less than 100, and the greater the *ROAV* value, the greater the contribution of the compounds to the overall flavor of the sample. It is generally believed that compounds with *ROAV* ≥ 1 are the characteristic flavor compounds of the analyzed sample group, and compounds with 0.1 ≤ *ROAV* < 1 have an important modification effect on the overall flavor of the sample group [15].

### 2.8. Data Analysis

To analyze the data, both one-way and two-way analyses of variance (ANOVA) were used. A *p*-value of less than 0.05 was regarded as statistically meaningful. Origin software version 2021 and GraphPad Prism version 9.4.1 were used for the statistical analysis.

## 3. Results and Discussion

### 3.1. Sensory Analysis of DSP Ham

**Figure 1:**
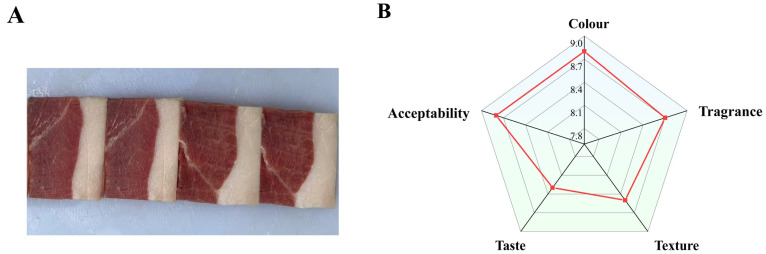
(**A**) DSP ham sample chart. (**B**) Sensory score radar chart of DSP ham.

As illustrated in Figure 1A, the muscle section of the DSP ham exhibited a pink hue, while the fat section was characterized by a creamy white and glossy appearance. This ham possesses a distinctive aroma, devoid of any unpleasant odors or rancidity. The muscle is both dry and compact, featuring delicate, tender meat with a smooth cut surface. The taste is described as mellow, fresh, and slightly salty, with a long-lasting aftertaste, making it highly acceptable overall. Figure 1B shows the sensory evaluation results for the 3-year-old DSP ham. On a 10-point scale, the results demonstrated that DSP ham had good flavor qualities with sensory scores of 8.6 ± 0.52 for flavor, 7.9 ± 0.57 for taste, 8.2 ± 0.79 for texture, 8.8 ± 0.42 for color, and 8.3 ± 0.48 for acceptability.

Zheng et al. [16] reported that Nuodeng ham exhibited the flavor, taste, and color characteristics of traditional air-dried ham, as determined by sensory evaluation. According to the current study’s findings, the compound’s curing agent significantly improved the ham’s pink hue and decreased its saltiness. Similarly, Li et al. [17] found that the texture, color, overall acceptability, and aroma scores of the ham samples obtained in the third year of maturation were significantly higher than those of samples evaluated in the first and second years (*p* < 0.05). This improvement may be due to changes in the flavor components over the extension of curing time.

Therefore, the DSP ham has excellent flavor and color, possibly due to the long fermentation time of 3 years. When exposed to acid, alkali, or enzymes, the protein in the muscle tissue breaks down into amino acids and small peptides. Meanwhile, the fat in the ham degrades, producing free fatty acids and other substances. Additionally, myoglobin is gradually oxidized to metmyoglobin. Concurrently, some reducing agents, such as vitamin C, interact with myoglobin to form a stable oxidation state, resulting in a bright red or rose color [18]. The sensory analysis results indicate that the flavor of 3-year-old DSP ham is exceptional. The formation of the ham’s excellent flavor was then further explored based on the free amino acids and lipid components in the ham.

### 3.2. Reveal the Characteristic Lipids of DSP Ham

#### 3.2.1. Lipid Composition of DSP Ham

**Figure 2:**
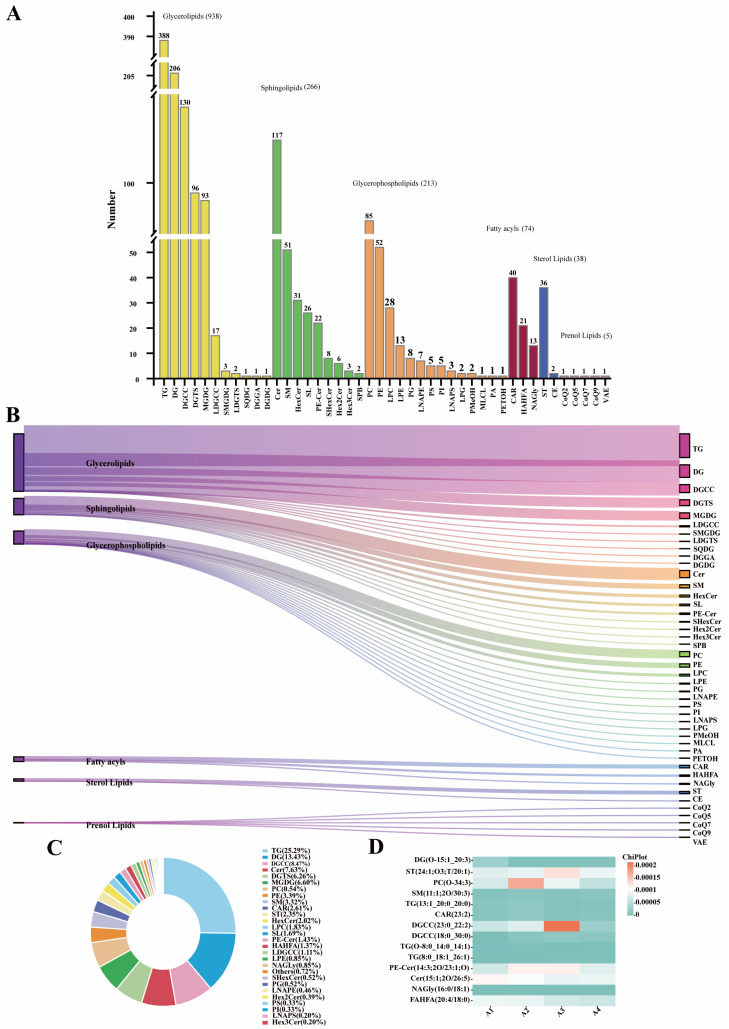
(**A**) Number of lipid species identified in DSP ham. (**B**) Sanger plot of all lipid types identified in DSP hams. (**C**) Percentage of all lipid types in DSP ham. (**D**) Heat map of relative content of key lipids in DSP ham.

The lipidomics technique known as UHPLC-OE-MS, which is non-targeted, was employed to determine the various lipid species and their respective quantities in three-year-old DSP ham. A total of 1534 lipid metabolites were identified from DSP ham in both positive- and negative-ion modes. As illustrated in Figure 2A,B, the lipids were classified into 6 primary categories and 44 subcategories. The predominant lipids identified in the 3-year-old dry-cured DSP ham included glycerides (GL; 938 species), sphingolipids (SP; 266 species), glycerophospholipids (GP; 213 species), fatty acyls (FA; 74 species), sterol esters (ST; 38 species), and isoprenol lipids (PR; 5 species). Overall, GL had the highest relative content and variety among the total lipid composition, followed by SPs and GP. TG were the most abundant, with 388 species identified. As shown in Figure 2C, these lipid molecules belonged to 44 different classes, including TG (25.29%), DG (13.43%), DGCC (8.47%), DGTS (6.26%), MGDG (6.06%), etc.

Zheng et al. [18] categorized the esters found in Norden ham into GP, GL, SP, FA, and other lipid classes. These lipid molecules were further classified into 16 subclasses, with TG representing the largest proportion, comprising 448 species. Guo et al. [19] identified 581 lipid metabolites in dry-cured mutton ham samples across various processing stages. These metabolites were categorized into 6 major categories and 21 subcategories. The total lipid content identified in DSP ham in the present study was greater than that found in mutton ham, likely due to differences in the raw materials used for ham production. The most abundant lipids identified in DSP ham were glycerol esters, followed by SP and GP. Glycerol esters are esters formed by glycerol and fatty acids, including monoesters, diesters, and triesters, among which triacylglycerol is the main form, and its decomposition to form free fatty acids represents the key step in flavor formation. The structural characteristics of SP allow for absorbing and retaining flavor compounds, thereby stabilizing the flavor of hams during storage and processing [20].

Additionally, the decomposition products of SP can serve as carriers for flavor substances, thereby enhancing flavor release. GP, which contains a relatively higher proportion of unsaturated fatty acids, yields a significant quantity of aldehydes and ketones upon oxidation. These compounds are crucial volatile flavor constituents, indicating that GP contributes substantially more to the overall flavor than other lipid classes. The TG content in the 3-year-old DSP ham was also high, which was similar to the above results. The degradation of lipids generates a significant quantity of volatile compounds. TG are the predominant lipids in both animals and plants, and their decomposition into free fatty acids is a crucial step for flavor development. As ham ages, natural and microbial enzymes catalyze the reaction of ester bond cleavage that results in fat hydrolysis, particularly triglyceride hydrolysis [21]. Triglyceride hydrolysis is dominated by lipase, the primary enzyme that converts triglycerides into glycerol and fatty acids [20]. Therefore, the hydrolysis of TG to yield free fatty acids is essential for enhancing the flavor profile of 3-year-old DSP ham.

#### 3.2.2. Characteristic Lipids in DSP Ham

During ham production, various enzymes and microorganisms cause the fat to break down into free fatty acids. These fatty acids are then oxidized to produce hydroperoxides, which further decompose to form the characteristic flavor substances and flavor precursors [22]. As shown in Figure 2D, analysis of the relative lipid contents of DSP ham identified 14 key lipids, including TG(13: 1_20: 0_20: 0), TG(O-8: 0_14: 0_14: 1), TG(8: 0_18: 1_26: 1), DG(O-15: 1_20: 3), PC(O-34: 3), SM(11: 1; 2O/30: 3), Cer(15: 1; 2O/26: 5), SHexCer(37: 1; 2O), FAHFA(20: 4/18: 0), NAGly(16: 0/18: 1), PE-Cer(14: 3; 2O/23: 1; O), DGCC(23: 0_22: 2), DGCC(18: 0_30: 0), and ST(24: 1; O3; T/20: 1). TG and DG account for the largest proportions in the GL class. The thermal degradation products of TG, including aldehydes, ketones, and furan compounds, can promote the Maillard reaction, generating pyrazine compounds that impart roasted and meaty flavors [23]. DG can promote the β-oxidation of fatty acids, thereby influencing the expression of genes related to lipid metabolism [24]. PC(O-34: 3) is the predominant phospholipid present in the membranes of all mammalian cells, as well as in protective membrane structures, playing a role in lipid metabolism throughout ham processing [25]. The phospholipid component of PC has specific antioxidant properties, which can inhibit the oxidation of lipids in ham and prolong the shelf life. SHexCer(37: 1; 2O) is a galactose sulfate ceramide. Previous studies have shown that foods rich in ceramides have a positive effect on human health [26]. SM(11: 1; 2O/30: 3) oxidation and hydrolysis reactions occur during ham processing to produce a series of volatile and non-volatile flavor compounds, which can be used as flavor precursors to further react with other components (such as amino acids), ultimately producing compounds with characteristic flavors [27]. Yuan et al. [28] discovered that the presence of SM in breast milk can stimulate brain and nervous system growth, as well as regulate intestinal bacteria. Cer is a key neutral lipid, acting as a precursor substance involved in lipid metabolism to form flavor as a decomposition product of SP [29]. FAHFA is a fatty acid hydroxyl fatty acid ester, serving as an important flavor precursor in ham, as it undergoes hydrolysis and oxidation reactions to produce a series of volatile and non-volatile compounds during food heating or fermentation [30]. In summary, 14 types of lipids were identified in DSP ham that make a significant contribution to its flavor.

### 3.3. Flavor Compounds of DSP Ham Revealed by UPLC-MRM-MS/MS and Flavoromics

#### 3.3.1. Non-Volatile Compounds in DSP Ham

There are many microorganisms involved in the process of ham fermentation. These microorganisms release proteases that break down the proteins found in ham. This process results in the degradation of proteins into smaller peptides, which are ultimately transformed into free amino acids. These amino acids are significant non-volatile flavor compounds in meat products. Therefore, the final content of free amino acids will affect the overall flavor of ham [31].

**Figure 3:**
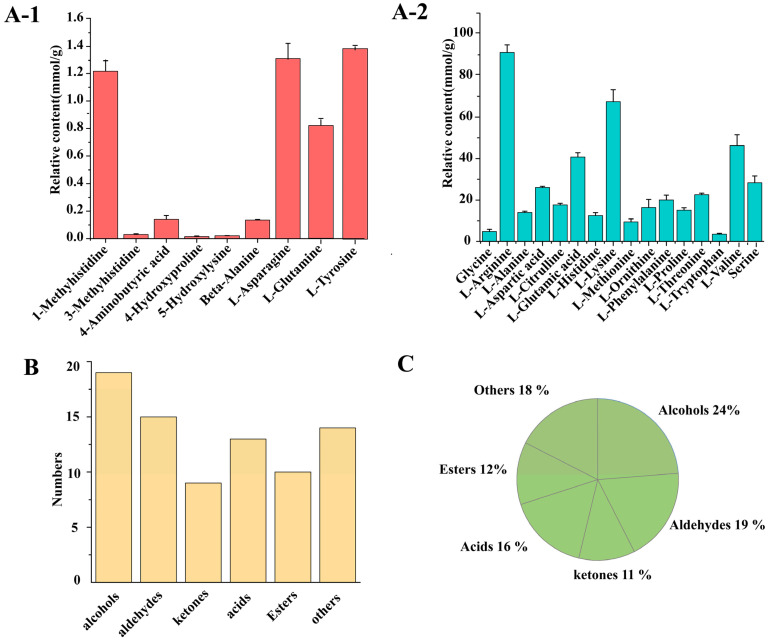
Flavor substances of DSP ham. (**A-1**,**A-2**) Free amino acids and their relative contents in DSP ham. (**B**) Types and quantities of volatile flavor substances identified in DSP ham. (**C**) Percentage of identified volatile flavor compounds.

UPLC-MRM-MS/MS was used to determine the free amino acids in DSP ham. Figure 3(A-1,A-2) illustrates that 25 distinct free amino acids were detected in 3-year-old DSP ham. The relative content of these 25 free amino acids varied greatly, and those with a relative content ≥ 40 mmol/g were selected as the key amino acids. The relative content of L-alanine was the highest, followed by L-lysine and L-valine. Amino acids mainly contribute to the umami, sweet, bitter, and neutral flavors in meat. L-alanine is a sweet amino acid, L-lysine results in a sweet and bitter taste, and L-valine is a neutral amino acid. Yan et al. [32] identified 16 types of amino acids in goose meat, with significant differences in amino acid content. Among them, bitter amino acids were the most abundant, followed by sweet and umami amino acids. In contrast, the content of sweet amino acids in DSP ham was found to be the highest; this difference may be due to the different varieties of meat. The interaction of various amino acids contributes to the final flavor of ham. Sweet amino acids and other flavor amino acids (such as umami amino acids and bitter amino acids) balance and influence each other, which together constitute the unique flavor of ham. The combined effect leads to an increased level of sweet amino acids, which play a significant role in enhancing the flavor of the ham.

#### 3.3.2. Composition of Volatile Compounds in DSP Ham

To further explore the flavor constituents of 3-year-old DSP ham, we analyzed the volatile compounds. A total of 80 volatile compounds were identified in the DSP ham after 3 years of fermentation (Table S1), including 15 aldehydes (19%), 19 alcohols (24%), 9 ketones (11%), 10 esters (12%), 13 acids (16%), and 14 other compound types (18%) (Figure 3C). As shown in Figure 3B, the most diverse substances were alcohols, followed by aldehydes. Deng et al. [33] detected 61 volatile flavor compounds from Jinhua ham, including 11 aldehydes, 5 acids, 4 alcohols, 9 esters, 3 ketones, 22 hydrocarbons, and 7 others. Li et al. [34] detected 130 flavor compounds in fish oil from the viscera of whitebait, including 14 alcohols, 37 aldehydes, 18 ketones, 6 esters, 27 hydrocarbons, 4 phenols, 11 heterocyclic compounds, 9 nitrogen-containing compounds, and 4 other compounds. Overall, volatile compounds were found to be more abundant in DSP ham than in Jinhua ham. This may be related to differences in the ham processing and storage conditions, resulting in different degrees of lipid oxidation.

#### 3.3.3. Characteristic Volatile Flavor Compounds in DSP Ham

During ham fermentation, fats hydrolyze to release a lot of free fatty acids, particularly polyunsaturated fatty acids, which are then oxidized by chains of free radicals to create hydroperoxides [35]. Aldehydes and other substances that contribute to common smells are produced by further cleaving these hydroperoxides. This is a crucial stage in the development of distinctive flavors [36]. In the late stage of fermentation, the water activity of the ham reduces and the presence of metal ions increases the oxidation of polyunsaturated fatty acids, thus promoting the synthesis of flavor compounds [37]. As highlighted above, aldehydes, alcohols, ketones, esters, acids, and other compounds were identified in DSP ham. However, each of these compounds has a different relative contribution to the flavor of ham, with some key volatile compounds (*ROAV* ≥ 1) showing a strong relationship to the flavor. As shown in Table 1, a total of 28 characteristic flavor compounds with *ROAV* ≥ 1 were detected from the samples, including 6 aldehydes, 8 alcohols, 7 esters, 2 acids, and 5 other compounds: valeraldehyde, hexanal, heptanal, octanal, nonanal, benzaldehyde, 1-octen-3-ol, n-heptanol, 2,6-dimethyl-4-heptanol, benzyl alcohol, (±)-6-methyl-5-hepten-2-ol, isopropanol, ethanol, sec-butanol, ethyl butyrate, ethyl 2-methylbutyrate, ethyl 3-methylbutyrate, ethyl hexanoate, phenethyl acetate, ethyl palmitate and methyl octanoate, myristic acid, palmitic acid, pyrazine, pyrrole, ethyl ketone, 1-(1H-pyrrole-2-yl)-, and caffeine.

Most alcohols are oxidized from lipids [38], but their sensory thresholds are higher. Among the detected flavor compounds, alcohols showed the greatest variety in DSP ham, which may be due to the long fermentation time of 3 years. That is, a longer period of oxidation of lipids resulted in the generation of more alcohol species in the DSP ham. Table 1 shows the 8 key alcohol compounds identified in the ham, including 1-octene-3-ol, n-heptanol, 2,6-dimethyl-4-heptanol, benzyl alcohol, (±)-6-methyl-5-hepten-2-ol, isopropanol, ethanol, and sec-butanol. 1-Octanol is formed by the degradation of unsaturated fatty acids [39]. The *ROAV* of 1-octene-3-ol in DSP ham was high (100), and the threshold value was low. Therefore, 1-octene-3-ol is an important flavor component that provides a distinct mushroom odor to the ham. The thresholds of other alcohols were very high, which may indicate a specific modification effect on the flavor of ham.

Aldehydes are related to lipid oxidation and are indispensable volatile compounds in meat [40]. Since the odor threshold of aldehydes is relatively low, the high content of aldehydes has a significant influence on the flavor of DSP ham. We identified 6 key aldehydes in the DSP ham, including pentanal, hexanal, heptanal, octanal, nonanal, and benzaldehyde. Deng et al. [33] found that aldehydes accounted for a large proportion of the total flavor substances in Jinhua ham, which were mainly branched (isovaleraldehyde), linear (hexanal, heptanal), and aromatic (benzaldehyde, phenylacetaldehyde) aldehydes. Similarly, aldehydes were identified as the second most abundant substances in the 3-year DSP ham.

Hexanal exhibited the highest *ROAV* among the aldehydes detected. At low concentrations, hexanal exhibits a grassy fragrance, whereas at elevated concentrations, it emits a fishy odor. Hexanal is primarily produced through the autoxidation of linoleic acid [41]. Shi et al. [42] found abundant hexanal in lard, which may also explain its high content in the ham of small-ear pigs of southern Yunnan, resulting in a significant contribution to the flavor of ham. Benzaldehyde has been characterized to produce an almond flavor, a bitter almond flavor, and a peach kernel flavor. Benzaldehyde is naturally present in many plant species. Some microorganisms can convert benzoic acid or benzyl alcohol into benzaldehyde [43]. Nonanal, which arises from the oxidation of oleic and linoleic acids, is noted for its plastic and fishy odor [44]. Octanal has a flavor characterized as sweet orange or honey, and it also produces a grassy flavor at low concentrations. Octanal is a volatile compound formed by lipid oxidation [45]. These aldehydes collectively contribute to the unique flavor of DSP ham.

Ester compounds have a fruity flavor and are mainly formed by the further esterification of the free fatty acids and alcohols produced by lipid oxidation. The plentiful alcohols present in DSP ham will undergo further esterification to produce additional esters, thereby enhancing their contribution to the ham’s flavor [46]. Ethyl acetate, ethyl 2-methylbutyrate, ethyl 3-methylbutyrate, ethyl hexanoate, phenethyl acetate, ethyl palmitate, and methyl octanoate were identified as the key ester compounds in DSP ham. Ethyl acetate has an intense fruity aroma, similar to the aroma of pineapple, banana, and pear, with some sweetness and slight sourness. Ethyl acetate is mainly formed by the esterification reaction between butyric acid, produced by fatty acid degradation, and alcohols, which can add to the flavor of ham [47]. Ethyl hexanoate has a pineapple-like fruit aroma and is the primary aroma substance of Luzhou-flavor liquor [48]. Acids function as the fundamental components in the synthesis of alcohols and esters. The primary source of acids is microbial metabolism and fat or amino acid degradation in the raw ham material [49]. Myristic acid and palmitic acid (both with *ROAV* ≥ 1) were identified in DSP ham. Due to their high thresholds, acids do not have a direct effect on food flavor [50]. Nevertheless, these two acid compounds may have a specific modification effect on the flavor profile of ham.

In summary, the hydrolysis and oxidation of lipids are two interdependent chemical events during ham fermentation, which together determine the production of taste compounds in ham. Among them, 28 essential flavor components were discovered, which, combined, comprised the flavor of DSP ham.

### 3.4. Flavor Formation Mechanism of DSP Ham

#### 3.4.1. Effect of Lipids on the Flavor of Ham

**Figure 4:**
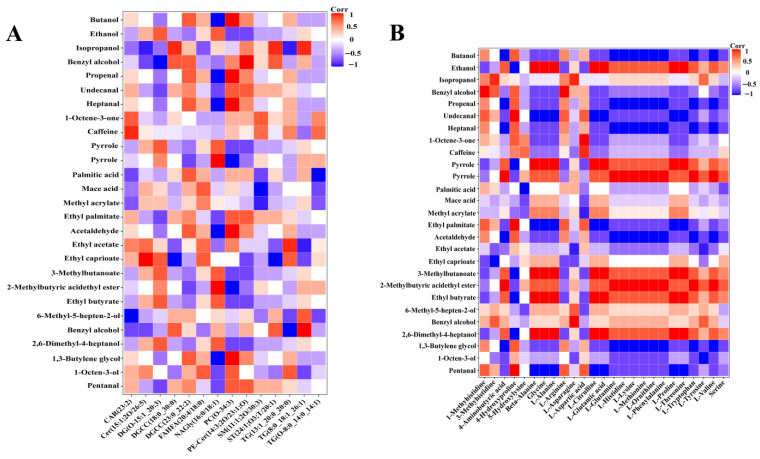
Correlation analysis. (**A**) Heatmap of correlation coefficients between key lipids and characteristic flavor substances identified in DSP ham. (**B**) Heatmap of correlation coefficients between free amino acids and characteristic flavor substances identified in DSP ham.

In addition to being flavor precursors, lipids also create volatile chemicals through oxidation and hydrolysis processes, which alter the ham’s flavor [51]. Therefore, we further analyzed the correlations between the 14 key lipids and 28 characteristic VOCs (*ROAV* ≥ 1) identified in DSP ham. The results of the Pearson correlation analysis are shown in Figure 4A. DG, NAGLy, and TG were positively correlated with pyrrole, ethyl 3-methylbutyrate, ethyl butyrate, ethanol, 2,6-dimethyl-4-heptanol, ethyl 2-methylbutyrate, pyrazine, and methyl octanoate in DSP ham. However, these three lipids were negatively correlated with ethyl palmitate, n-valeraldehyde, n-octanal, n-heptanal, sec-butanol, n-nonanal, n-hexanal, and n-heptanal. This indicates that the degradation of DG, NAGLy, and TG may have a strong relationship with the formation of these flavor components. ST, DGCC, PC, and PE-cer were negatively correlated with pyrrole, ethyl 3-methylbutyrate, ethyl butyrate, ethanol, and 2,6-dimethyl-4-heptanol, whereas these lipids were positively correlated with ethyl palmitate, n-valeraldehyde, n-octanal, n-heptanal, sec-butanol, n-nonanal, n-hexanal, and n-heptanal. DG can generate free fatty acids, monoacylglycerol, and short-chain volatile compounds such as hexanal and nonanal during heating or enzymatic hydrolysis [52].

Nonanal, hexanal, and 1-octene-3-ol, which play an important role in the flavor of DSP ham (*ROAV* ≥ 1), were found to be negatively correlated with TG and NAGLy. TG is the precursor of saturated, monounsaturated, and polyunsaturated fatty acids, which will degrade to produce flavor substances such as hexanal, nonanal, and octanal. Liu et al. [53] found that TG is a key lipid associated with key VOCs identified from precooked Chinese stewed beef. Consistently, TG was identified as an important flavor precursor in DSP ham in the present study. PC is phosphatidylcholine, which produces a variety of flavor substances during heating, oxidation, and other processes, including aldehydes, ketones, and alcohols [54]. Phospholipids are considered to be the key lipids that produce aroma compounds [55]. In this study, PC was strongly negatively correlated with ethanol, 2,6-dimethyl-4-heptanol, ethyl 2-methylbutyrate, and pyrazine.

#### 3.4.2. Effect of Free Amino Acids on the Flavor of Ham

Free amino acids significantly influence the formation of volatile compounds due to their inherent flavor characteristics. The primary source of amino acids in ham is the breakdown of proteins and peptides by enzymes during fermentation and ripening. The proportion of various amino acids impacts the flavor of dry-cured ham [56]. A total of 25 free amino acids and 28 characteristic VOCs (*ROAV* ≥ 1) were selected for correlation analysis, the results of which are shown in Figure 4B. L-Valine, L-tryptophan, L-phenylalanine, L-ornithine, L-methionine, L-lysine, L-histidine, 4-aminobutyric acid, L-glutamine, L-threonine, L-proline, L-glutamic acid, L-citrulline, L-alanine, beta-alanine, glycine, and pyrrole were positively correlated with ethyl 3-methylbutyrate, ethyl butyrate, ethanol, 2,6-dimethyl-4-heptanol, ethyl 2-methylbutyrate, pyrazine, ethyl hexanoate, methyl octanoate, and benzyl alcohol, whereas these amino acids were negatively correlated with benzaldehyde, ethyl palmitate, n-pentanal, n-octanal, n-heptanol, sec-butanol, n-nonanal, n-hexanal, n-heptanal, 1-octen-3-ol, phenylethyl acetate, caffeine, and 1-octen-3-one. These results suggest that characteristic flavor compounds identified in DSP ham, such as nonanal, hexanal, 1-octen-3-ol, and 1-octen-3-one, are significantly related to the concentrations of these amino acids.

Amino acids act as building blocks for flavor compound formation, and their breakdown improves the flavor constituents [57]. Larrea et al. [58] noted that substantial proteolysis takes place throughout the processing of dry-cured ham, resulting in a gradual buildup of small peptides and free amino acids. Lipid oxidation products and free amino acids are degraded by Strecker to generate Strecker aldehydes and pyrazine compounds to enhance nut and chocolate fragrances [59]. The methyl-branched aldehydes identified in mature turkey hams primarily originate from the Strecker degradation of free amino acids, including L-valine, isoleucine, and leucine [60]. Zhu et al. [61] investigated the role of the Maillard reaction in the formation of flavor compounds in Jinhua ham, revealing that glucose reacts with L-lysine to produce 10 volatile compounds. L-methionine is degraded by sulfur-containing amino acids to produce flavor compounds such as nonanal and 1-octen-3-one [62]. L-phenylalanine can be degraded via the Strecker degradation process to yield aldehydes, including benzaldehyde [63]. L-glutamine can be hydrolyzed to glutamic acid through the action of glutaminase, which is one of the primary sources of the umami taste [64]. Our analysis indicates that the majority of flavor compounds in DSP ham originate from free amino acids, which serve as flavor precursors influencing the overall flavor profile of the ham.

**Figure 5:**
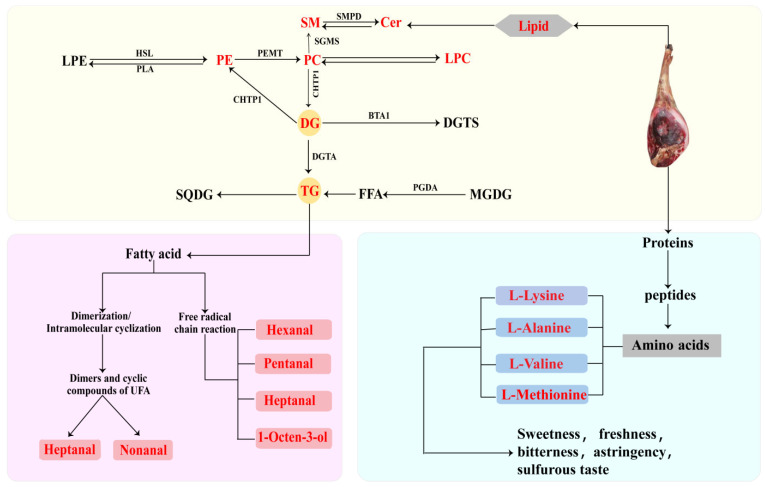
Proposed transformation pathways of lipid molecules and free amino acids in DSP ham.

The potential transformation pathways of lipid molecules and free amino acids in DSP ham are illustrated in Figure 5. Overall, lipids identified through lipidomics, including DG, TG, and PC, may play a crucial role in the formation of VOCs that contribute to the flavor of DSP ham. Using the glycerophospholipid pathway, phospholipase A2 (PLA2) hydrolyzes glycerophospholipids (such as PC and phosphatidylethanolamine (PE)) to provide lysophospholipids and free polyunsaturated fatty acids (PUFA) [65]. It is more likely that unsaturated fatty acids will undergo oxidation when they hydrolyze into free fatty acids and hydroperoxides, which can then be further broken down into aldehydes and other volatile compounds [36]. Notably, key volatile compounds such as hexanal, pentanal, heptanal, and 1-octene-3-ol were identified in DSP ham.

## 4. Conclusions

This study used lipidomics and flavoromics to determine the mechanism of flavor production in 3-year-old DSP ham. The results showed that there were no noticeable defects in the sensory quality and that overall acceptance was good. The ham was found to contain 1534 lipids, 80 volatile taste compounds, and 25 free amino acids, of which 14 were characteristic lipids and 28 were important flavor compounds. We discovered a negative correlation between TG and DG and a few aldehydes. Additionally, n-nonenal, n-hexanal, n-heptanal, and 1-octen-3-ol showed negative relationships with L-valine, L-methionine, L-lysine, and L-alanine. In conclusion, the mutually reinforcing metabolism of lipids and amino acids controls the generation of tastes in DSP ham. A useful resource for the creation of premium ham products is this study. Future research will focus on the role of microbial communities during the fermentation of DSP hams to investigate their effect on ham quality.

## Figures and Tables

**Table 1:** Characteristic volatile flavor compounds in DSP ham.

NO.	Compounds	Threshold (µg/kg)	Relative Content (%)	*ROAV*
**1**	Pentanal	12.00	1.2274	2.3998
**2**	Hexanal	4.00	4.6181	27.0866
**3**	Heptanal	3.00	0.9151	7.1566
**4**	Octanal	1.40	0.6394	10.7155
**5**	Nonanal	1.00	0.1004	2.3544
**6**	Benzaldehyde	3.00	1.6205	12.6729
**7**	Isopropyl Alcohol	40,000.00	0.0010	5.7935 × 10^−7^
**8**	Ethanol	8.00	1.1285	3.3096
**9**	2-Butanol	43,000.00	0.0324	1.7704 × 10^−5^
**10**	1-Octen-3-ol	1.00	4.2624	100.0000
**11**	1-Heptanol	3.00	0.5823	4.5535
**12**	4-Heptanol, 2,6-dimethyl-	1300.00	0.0020	3.6283 × 10^−5^
**13**	Benzyl alcohol	1.20	0.0678	1.3258
**14**	5-Hepten-2-ol,6-methyl-	2000.00	0.0006	7.1909 × 10^−6^
**15**	1-Octen-3-one	0.05	0.0627	29.4274
**16**	Butanoic acid, ethyl ester	0.10	0.0104	2.4337
**17**	Butanoic acid, 2-methyl-, ethyl ester	0.01	0.0024	5.7434
**18**	Butanoic acid, 3-methyl-, ethyl ester	0.01	0.0033	7.7816
**19**	Hexanoic acid, ethyl ester	0.30	0.0375	2.9309
**20**	Acetic acid, 2-phenylethyl ester	3000.00	0.0031	2.4501 × 10^−5^
**21**	Hexadecanoic acid, ethyl ester	2000.00	0.0064	7.5155 × 10^−5^
**22**	Octanoic acid, methyl ester	200.00	0.0004	4.5969 × 10^−5^
**23**	Tetradecanoic acid	10,000.00	0.0008	1.9034 × 10^−6^
**24**	n-Hexadecanoic acid	10,000.00	0.0163	3.8225 × 10^−5^
**25**	Pyrazine	175,000.00	0.0449	6.0243 × 10^−6^
**26**	Pyrrole	20,000.00	0.0133	1.5546 × 10^−5^
**27**	Ethanone, 1-(1H-pyrrol-2-yl)-	170,000.00	0.0046	6.3212 × 10^−7^
**28**	Caffeine	29,000.00	0.0003	2.4773 × 10^−7^

## Data Availability

The original contributions presented in this study are included in the article/Supplementary Material. Further inquiries can be directed to the corresponding authors.

## Supplementary Materials

The following supporting information can be downloaded at: https://www.mdpi.com/article/10.3390/foods14173098/s1. Table S1: Volatile flavor compounds in DSP ham.
