# Supplementary material for: Formation Mechanism of Characteristic Flavor Substances in 3-Year-Old Diannan Small-Ear Pig Ham: Lipidomics and Flavoromics Study
Source: Foods. 2025 Sep 4;14(17):3098. doi: 10.3390/foods14173098 (PMC12428617; doi:10.3390/foods14173098)
Supplement: Supplementary file 1 [file foods-14-03098-s001.zip › foods-3802857-supplementary.pdf]

Table S1. Volatile flavor compounds in DSP ham.

Types of compounds	NO.	Compounds	Threshold (μg/kg)	Comparative content(%)	ROAV
Aldehydes	A1	Butanal, 2-methyl-	1.00	0.0194	0.4542
	A2	Pentanal	12.00	1.2274	2.3998
	A3	Hexanal	4.00	4.6181	27.0866
	A4	2-Pentenal, (E)-	1.50	0.0031	0.0491
	A5	Heptanal	3.00	0.9151	7.1566
	A6	2-Butenal, 2-methyl-	500.00	0.0660	0.0031
	A7	Octanal	1.40	0.6394	10.7155
	A8	2-Heptenal, (E)-	13.00	0.0910	0.1642
	A9	Nonanal	1.00	0.1004	2.3544
	A10	2-Octenal, (E)-	3.00	0.0202	0.1583
	A11	Decanal	9.00	0.0071	0.0186
	A12	Benzaldehyde	3.00	1.6205	12.6729
	A13	2,6-Nonadienal, (E,E)-	0.09	0.0029	0.7682
	A14	Benzeneacetaldehyde	4.00	0.0025	0.0149
	A15	2(3H)-Furanone, dihydro-5-pentyl-	7.00	0.0515	0.1725
Alcohols	B1	Isopropyl Alcohol	40000.00	0.0010	5.7935×10^{-7}
	B2	Ethanol	8.00	1.1285	3.3096
	B3	2-Butanol	43000.00	0.0324	1.7704×10^{-5}
	B4	2-Nonen-1-ol, (Z)-	130.00	0.0016	0.0003
	B5	1-Propanol, 2-methyl-	360.00	0.0145	0.0009
	B6	2-Butanol,	410.00	0.0284	0.0016

		3-methyl-			
	B7	1-Butanol	500.00	0.0389	0.0018
	B8	1-Penten-3-ol	400.00	0.1792	0.0105
	B9	1-Butanol, 3-methyl-	250.00	0.2375	0.0223
	B10	1-Pentanol	120.00	0.9783	0.1913
	B11	2-Undecanol	8.60	0.0196	0.0536
	B12	1-Octen-3-ol	1.00	4.2624	100
	B13	1-Heptanol	3.00	0.5823	4.5535
	B14	4-Heptanol, 2,6-dimethyl-	1300.00	0.0020	3.6283×10^{-5}
	B15	Linalool	4.00	0.0039	0.0229
	B16	1-Octanol	42.00	0.5350	0.2988
	B17	Benzyl alcohol	1.20	0.0678	1.3258
	B18	Phenylethyl Alcohol	1000.00	0.0295	0.0007
	B19	5-Hepten-2-ol,6-methyl-	2000.00	0.0006	7.1909×10^{-6}
ketone	C1	2-Pentanone	70.00	0.6936	0.2325
	C2	2,3-Pentanedione	20.00	0.0379	0.0445
	C3	2-Heptanone	20.00	0.1059	0.1243
	C4	3-Octanone	21.00	0.0210	0.0234
	C5	2-Octanone	41.00	0.0362	0.0207
	C6	1-Octen-3-one	0.05	0.0627	29.4274
	C7	5-Hepten-2-one,6-methyl-	50.00	0.2515	0.1180
	C8	2-Nonanone	5.00	0.0332	0.1556
	C9	Acetophenone	65.00	0.0159	0.0058
Ester	D1	Butanoic acid, ethyl	0.10	0.0104	2.4337

		este			
	D2	Butanoic acid, 2-methyl-, ethyl ester	0.01	0.0024	5.7434
	D3	Butanoic acid, 3-methyl-, ethyl ester	0.01	0.0033	7.7816
	D4	Pentanoic acid, ethyl ester	1.50	0.0025	0.0394
	D5	Hexanoic acid, ethyl ester	0.30	0.0375	2.9309
	D6	1-Butanol, 2-methyl-, acetate	5.00	0.0393	0.1843
	D7	Octanoic acid, ethyl ester	5.00	0.0132	0.0620
	D8	Acetic acid, 2-phenylethyl ester	3000.00	0.0031	2.4501×10^{-5}
	D9	Hexadecanoic acid, ethyl ester	2000.00	0.0064	7.5155×10^{-5}
	D10	Octanoic acid, methyl ester	200.00	0.0004	4.5969×10^{-5}
Acid	E1	Formic acid	62.50	0.0014	0.0005
	E2	Propanoic acid	5000.00	0.0699	0.0003
	E3	Propanoic acid, 2-methyl-	10.00	0.0517	0.1214
	E4	Butanoic acid	240.00	1.7279	0.1689
	E5	Butanoic acid, 3-methyl-	190.00	0.7777	0.0960
	E6	Pentanoic acid	940.00	0.3361	0.0084

	E7	Hexanoic acid	93.00	3.4726	0.8760
	E8	Heptanoic acid	640.00	0.1195	0.0044
	E9	Octanoic acid	910.00	0.4586	0.0118
	E10	Nonanoic acid	3000.00	0.0498	0.0004
	E11	n-Decanoic acid	2200.00	0.0624	0.0007
	E12	Tetradecanoic acid	10000.00	0.0008	1.9034×10^{-6}
	E13	n-Hexadecanoic acid	10000.00	0.0163	3.8225×10^{-5}
Other	F1	Pyridine	820.00	0.0083	0.0002
	F2	Pyrazine	175000.00	0.0449	6.0243×10^{-6}
	F3	Thiazole	3100.00	0.0420	0.0003
	F4	Pyrazine, methyl-	60.00	0.1108	0.0433
	F5	Pyrazine,2,3-dimethyl-	800.00	0.0531	0.0016
	F6	Pyrazine, 2-ethyl-6-methyl-	100.00	0.0235	0.0055
	F7	Pyrazine, trimethyl-	400.00	0.0128	0.0008
	F8	Pyrazine,tetramethyl-	1000.00	0.0137	0.0003
	F9	Pyrrole	20000.00	0.0133	1.5546×10^{-5}
	F10	2-Isobutylthiazole	2.00	0.0300	0.3518
	F11	2-Acetylthiazole	4.00	0.0124	0.0729
	F12	Ethanone, 1-(1H-pyrrol-2-yl)-	170000.00	0.0046	6.3212×10^{-7}
	F13	Indole	140.00	0.0022	0.0004
	F14	Caffeine	29000.00	0.0003	2.4773×10^{-7}
